# Low-toxic herbicides Roundup and Atrazine disturb free radical processes in Daphnia in environmentally relevant concentrations

**DOI:** 10.17179/excli2022-4690

**Published:** 2022-03-09

**Authors:** Viktor Husak, Tetiana Strutynska, Nadia Burdyliuk, Anzhelika Pitukh, Volodymyr Bubalo, Halina Falfushynska, Olha Strilbytska, Oleh Lushchak

**Affiliations:** 1 Department of Biochemistry and Biotechnology, Vasyl Stefanyk Precarpathian National University, 57 Shevchenka Str., Ivano-Frankivsk, 76018, Ukraine; 2 Laboratory of Experimental Toxicology and Mutagenesis, L.I. Medved's Research Center of Preventive Toxicology, Food and Chemical Safety, MHU, Kyiv, Ukraine; 3 Ternopil Volodymyr Hnatiuk National Pedagogical University, M. Kryvonosa, 2, Ternopil, 46027, Ukraine; 4 Research and Development University, 13a Shota Rustaveli Str., Ivano-Frankivsk, 76018, Ukraine

**Keywords:** Roundup, Atrazine, Daphnia, oxidative stress, toxicity

## Abstract

The use of glyphosate-based Roundup and triazine herbicide Atrazine has increased markedly in last decades. Thus, it is important to evaluate toxic effects of these herbicides to non-targeted organisms such as zooplankton to understand their safety toward aquatic ecosystems. In the current study, we performed Daphnia toxicity tests based on lethality to identify LC_50_ that provides acute aquatic toxicity classification criteria. LC_50_ for Roundup exposure for 24 hours was found to be 0.022 mg/L and 48 hours - 0.0008 mg/L. Atrazine showed LC_50_ at concentrations of 40 mg/L and 7 mg/L for 24 and 48 hours, respectively. We demonstrated that exposure to ecologically relevant concentrations of Roundup or Atrazine decreases lipid peroxidation and protein thiol levels, however caused increase in carbonyl protein and low-molecular-weight thiols content. Moreover, the herbicide treatments caused increase of superoxide dismutase activity. Our data suggest that at very low concentrations Roundup and Atrazine disturb free radical processes in *D. magna*.

## Introduction

The antiweed herbicides Roundup and Atrazine are among most commonly used treatments in the world. Research surrounding safety of these herbicides has been controversial since Roundup causes varied diseases from cancer to autism (Wang et al., 2019[51]; Pu et al., 2020[43]), and Atrazine causes breast, ovarian, uterine cancers as well as leukemia and lymphoma (Pathak and Dikshit, 2011[38]). However, reports provided by industry claim they have very low toxicity toward non-target organisms. An active ingredient in Roundup is organophosphate glyphosate, N-(phosphonomethyl)glycine, in the form of its isopropylamine salt, with additional formulations containing so-called "inert" ingredients in different proportions, that are believed to be more toxic than glyphosate (Phyu et al., 2004[42]). The mechanism of glyphosate toxicity in non-target organisms is mostly unknown, but it may cause uncoupling of oxidative phosphorylation (Peixoto, 2005[40]). Atrazine, a triazine herbicide, that consists of a triazine ring, along with five nitrogen and one chlorine atoms, is used in the form of emulsifiable concentrate, soluble powder and granules. Investigation of the potential toxicity of Roundup and Atrazine on various model organisms may help to elucidate their toxicity and the defense pro-survival mechanisms. 

The unique biological features make *Daphnia* a very suitable model system for toxicological evaluations. Different parameters of toxicity, especially those based on lethality, are mainly used in toxicological studies on *Daphnia*. Toxicity parameter, LC_50_ is referred as a concentration that causes mortality of 50 % of individuals in a period of observation time. It can be used as the first screening method for the assessment of the lethal toxicity (Guilhermino et al., 2000[16]). Additionally, the toxic effects of tested drugs could be assessed using enzymatic and non-enzymatic biomarkers of toxicity in concentrations below those that cause mortality. Since some toxicants may cause oxidative stress, the toxicity for *D. magna* can be assessed with an array of oxidative stress markers including activities of glutathione-*S*-transferase, catalase, superoxide dismutase, glutathione reductase (Dionísio et al., 2020[8]; Nkoom et al., 2019[33]) and oxidation-end products of lipids such as thiobarbituric acid reactive substances (Lushchak et al., 2004[26]).

Growing evidence suggests that organismal exposure to commercial herbicide formulations may perturb free radical processes and inhibit the mitochondrial respiratory chain (Bailey et al., 2018[2]). The molecular mechanisms of oxidative stress induction by glyphosate-based Roundup and Atrazine are well characterized. Uncoupling of mitochondrial oxidative phosphorylation may be a major effect of herbicide intoxication (Pathak et al., 2011[38]; Peixoto, 2005[40]). The impaired mitochondrial function caused by glyphosate-based herbicides can be related to increased ROS generation (Bailey et al., 2018[2]; Gomes and Juneau, 2016[14]). Atrazine induces mitochondrial dysfunction by disruption of membrane potential and depletion of intracellular ATP levels (Liu et al., 2013[24]).

This study aimed to evaluate the effects of Roundup and Atrazine in *D. magna*, affecting the parameters of survival. This is the first study on the effects of Roundup and Atrazine on oxidative stress indicators in *D. magna* exposed to concentrations that did not cause mortality. As reported herein, this research provided important information on the physiological and biochemical effects associated with the exposure of aquatic organisms to Roundup or Atrazine, focusing on disturbance of free radical processes caused by glyphosate-based and triazine herbicides and antioxidant response. The data obtained can be used to improve environmental risk assessments. 

## Materials and Methods

### Reagents

Phenylmethylsulfonyl fluoride (PMSF), ethylenediamine-tetraacetic acid (EDTA), xylenol orange, cumene hydroperoxide, ferrous sulphate, *N,N,N',N'*-tetramethylethylenediamine were purchased from Sigma-Aldrich Corporation (USA). Roundup and Atrazine were purchased from Bayer AG (Germany) and Shanghai Sinogreatland Industrial Co., Ltd. (China), respectively. All other reagents were of analytical grade. 

### Animals and experimental conditions

*Daphnia magna* Straus is kept in the laboratory for more than 10 years. Water fleas were fed baker yeasts (0,25 million cells per L) and half of the water was changed twice a week. Water parameters were 20 ± 2 °C, pH 7.6-7.8, 7.2-7.6 mg/L of O_2_. The photoperiod was 16h light:8h dark. Experiments were carried out in a static mode, under the same conditions, but with the addition of the commercial herbicides Roundup which contains glyphosate [N-(phosphonomethyl)glycine] or Atrazine at a concentration of 550 or 500 g/L, respectively. Groups of forty 7-8 days old organisms were placed in 3L glass jars with different nominal concentrations of either Roundup or Atrazine herbicides: 0.1; 0.5; 1 and 5 µg/L. Herbicide concentrations used in this work were selected based on an LC_50_^24 ^value (half-lethal dose after 24 h exposure) for Roundup (0.022 mg/L) and for Atrazine exposure (40 mg/L), which were defined according to ISO standard (OECD, 2004[35]; Olorunsogo et al., 1979[36]). Water fleas were treated for 24 h (no mortality was observed at the studied concentrations). Studied parameter were measured in at least four biological replicates. 

### Acute toxicity tests

All experiments were performed according to the standard procedure (Olorunsogo et al., 1979[36]) for determining 24 and 48 h LC_50_ for *D. magna*. Water fleas were not fed during the tests. Twenty neonates (~ 24 hours old) per treatment in groups of five per 250 mL of test solution were used. Temperature and photoperiod were as described above. Oxygen concentrations and pH levels were determined at 0, 24, and 48 h time points. 

Groups of twenty organisms (in quadruplicate with 5 organisms of every instars per replicate) were placed in glass jars with different nominal concentrations of Roundup or Atrazine herbicides: 0.0005; 0.001; 0.005; 0.01; 0.02; 0.03; 0.05; 0.1; 0.5; 1; 3; 5; 10; 30; 50 and 100 mg/L. The animals in a control group were maintained in the same manner but without the addition of drug into the water. Death was recognized as immobilization for 15 s after stimulation by a bright light. After exposure, animals were dried by filter paper and used for analysis. 

### Determination of oxidative stress indices 

Lipid peroxide (LOOH) content was assayed by the FOX (ferrous-xylenol orange) method (Hermes-Lima et al., 1995[17]). Aliquots of the supernatants were used for the assay as described previously (Lushchak et al., 2005[30]). The content of LOOH was expressed as nanomoles of cumene hydroperoxide equivalents per gram wet mass of tissue.

Carbonyl groups of proteins (CP) were determined as described previously (Lenz et al., 1989[23]). Animals were crashed by homogenization (1:10, w:v in 50 mM potassium phosphate buffer, pH 7.0, 0.5 mM EDTA, 1 mM PMSF) and centrifuged (8,000 g, 15 min, 4 °C). Supernatants were removed and 0.25 ml aliquots were mixed with 0.25 ml of 40 % trichloroacetic acid (TCA) (final concentration 20 %) and centrifuged (5,000 g, 5 min, 21 °C). CP levels were measured in the resulting pellets by reaction with 2,4-dinitrophenylhydrazine, leading to the formation of dinitrophenylhydrazones (Lenz et al., 1989[23]). Values are expressed as nanomoles of carbonyl groups per milligram protein (nmol mg protein^-1^).

Free thiols were measured spectrophotometrically by the Ellman procedure (Ellman, 1959[9]). Total thiol concentration was measured in supernatants prepared as for the CP assays (Lushchak and Bagnyukova, 2006[29]). For determination of low-molecular-mass thiols (L-SH), aliquots of supernatants were mixed with TCA to reach a final TCA concentration of 10 %, centrifuged (16,000 g, 5 min, 21 °C) to remove pelleted protein and the final supernatants were used for the assay. Thiol concentrations were expressed as micromoles of SH-groups per gram tissue wet mass. The high-molecular-mass or protein thiol (P-SH) content was calculated by subtracting the L-SH from the total thiol concentration.

### Assay of enzyme activities and protein concentration

Supernatants were prepared as described above for CP. The activities of antioxidant enzymes including superoxide dismutase (SOD) and catalase were measured as described previously (Lushchak et al., 2005[30]). One unit of SOD activity was defined as the amount of enzyme (per mg of protein) that inhibited a quercetin oxidation reaction by 50 % of maximal inhibition. Inhibition values for SOD activity were calculated using an enzyme Kinetics computer program (Brooks, 1992[6]). One unit (U) of catalase activity is defined as the amount of enzyme that consumed 1 μmol of hydrogen peroxide per minute expressed per milligram of soluble protein.

Soluble protein concentrations were measured by the Coomassie blue method (Bradford, 1976[5]) using bovine serum albumin as a standard. 

### Statistics

The mortality data were used to calculate 24 and 48 h the median lethal concentration (LC_50_) by Probit analysis (Stephan, 1977[47]). Data are presented as means ± S.E.M. Statistical analyses were carried out in Prism 7 (GraphPad Software, USA) using analysis of variance ANOVA followed by the Dunnett's test to compare multiple experimental treatments to the control value with *p* < 0.05 as statistically significant.

## Results

### Mortality

**Figure 1:**
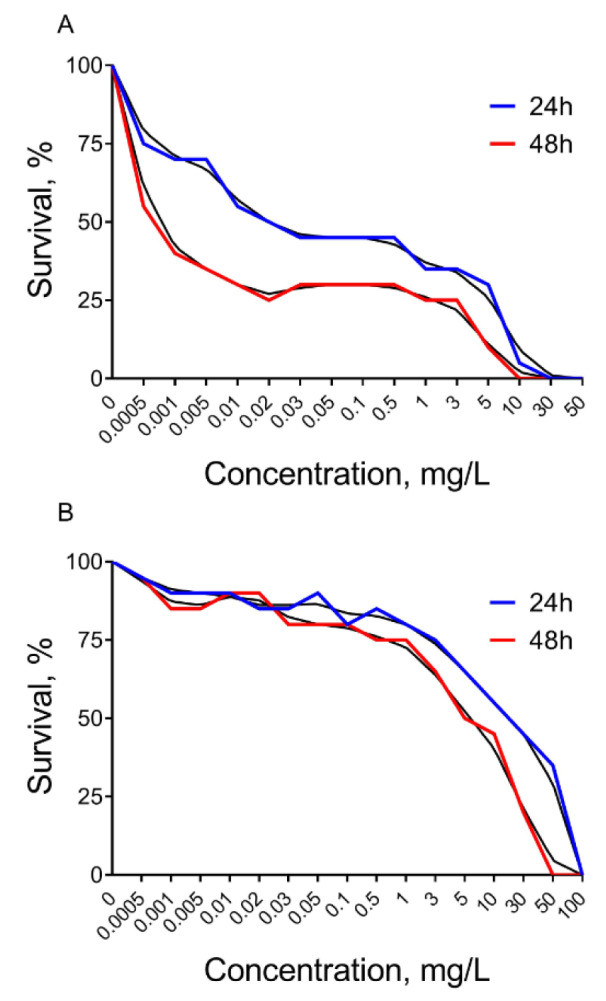
The mortality rate of *Daphnia magna* under 24 and 48 hours of exposure to Roundup (A) and Atrazine (B) treatments 0.0005-100 mg/L

Roundup herbicide causes *Daphnia* mortality at very low concentrations. According to a Probit test, it was found that Roundup at a concentration of 0.022 mg/L led to 50 % *Daphnia *mortality during 24 h (LC_50_^24 ^= 0.022 mg/L) (Figure 1A(Fig. 1)). We observed 50 % of dead individuals after Roundup treatment for 48 hours at a concentration of 0.0008 mg/L (LC_50_^48^ = 0.0008 mg/L). We also found complete mortality at concentrations of 30 mg/L (LC_100_^24^ ≥ 30 mg/L) and 10 mg/L (LC_100_^48^ ≥ 10 mg/L) during 24 and 48 hours of Roundup exposure, respectively (Figure 1A(Fig. 1)).

Atrazine herbicide was found to be less toxic on *Daphnia*. According to a Probit test 50 % of *Daphnia* mortality was observed under herbicide exposure at a concentration of 40 mg/L for 24 hours (LC_50_^24^ = 40 mg/L) (Figure 1B(Fig. 1)). Atrazine kills 50 % of the *Daphnia* individuals after 48 hours at a concentration of 7 mg/L (LC_50_^48^ = 7 mg/L). Exposure to concentrations of 100 and 50 mg/L of this herbicide resulted in the complete mortality of *Daphnia* population within 24 hours (LC_100_^24^ ≥ 100 mg/L) and 48 hours (LC_100_^48^ ≥ 50 mg/L), respectively.

### Oxidative stress markers 

Based on the LC_50_^24^ for Roundup and Atrazine, which was defined earlier (paragraph “Mortality”), we tested the concentrations of herbicide that was significantly lower than LC_50_^24^ and did not cause the death of *Daphnia* aged 7-8 days. The following concentrations of drugs were tested: 0.1; 0.5; 1 and 5 μg/L.

**Figure 2:**
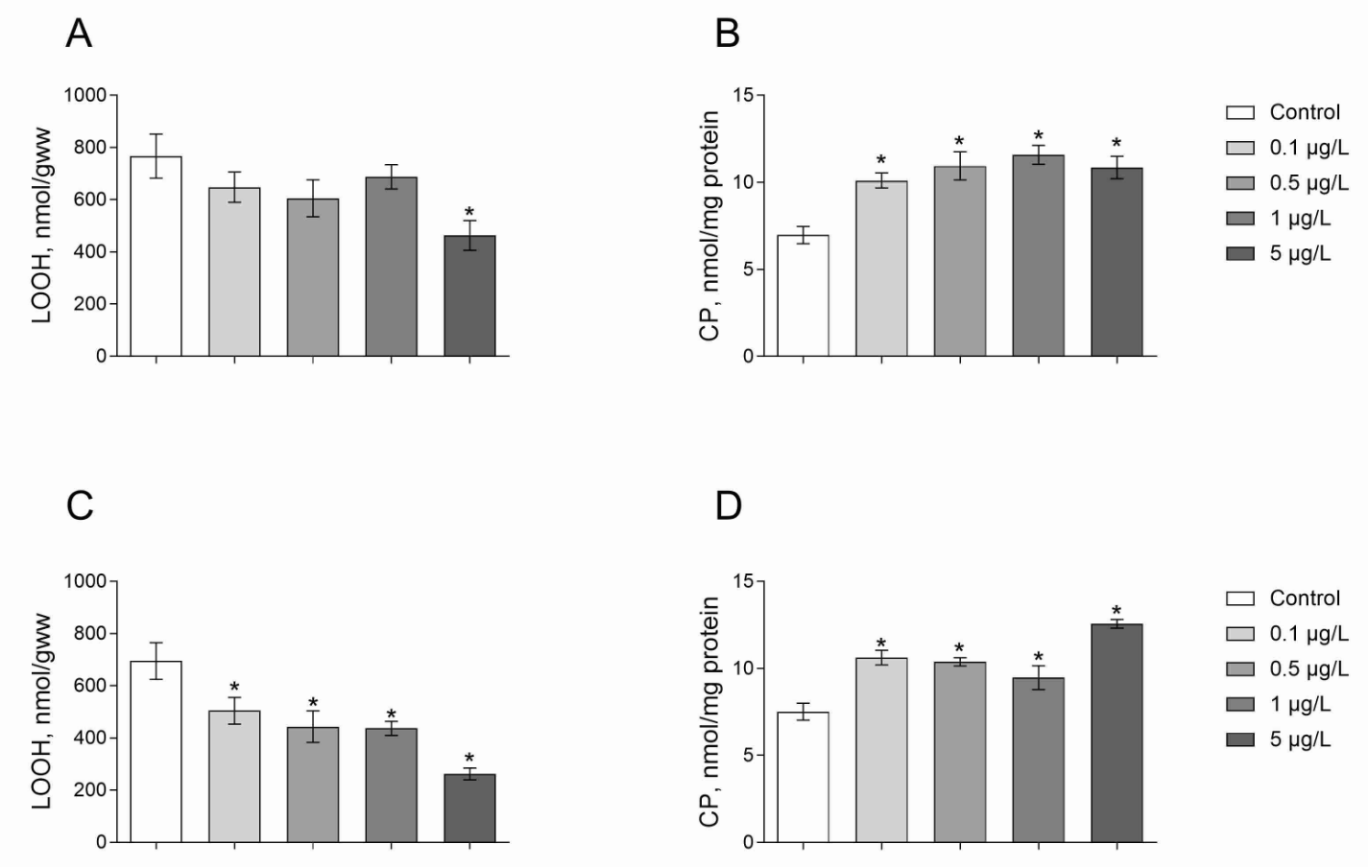
Levels of lipid peroxides and carbonyl proteins in *D. magna* exposed to Roundup (A, B) or Atrazine (C, D) for 24 hours. Values are expressed as mean ± S.E.M. (n = 4-6). *Significantly different with *p* < 0.05 (ANOVA followed by Dunnett's multiple comparison tests)

We measured the level of oxidatively modified molecules including protein- and low-molecular-mass thiol-group-containing compounds, and lipid peroxide contents in *Daphnia* as a measure of oxidative homeostasis in response to different concentrations of herbicides. We found that the level of lipid peroxides (LOOH) was largely decreased under Roundup exposure. The content of LOOH decreased by 40 % under the Roundup exposure at a concentration of 5 μg/L as compared to the control (Figure 2A(Fig. 2); Dunnett's test,* p* = 0.015). Atrazine treatment for 24 hours at all concentrations tested led to a lower LOOH level by 27-62 % as compared to animals of the control group (Figure 2B(Fig. 2); Dunnett's test, *p* < 0.05). 

ROS and their derivatives can induce the formation of additional carbonyl groups in proteins (CP). Given this, CP content serves as a reliable indicator for the assessment of oxidative damage. We found that *Daphnia* treatment with Roundup led to an increase in CP content as compared with the control group (Figure 2C(Fig. 2)). Indeed, 24 hours of exposure to Roundup at the concentrations of 0.1, 0.5, 1 and 5 μg/L results in 45-65 % higher CP content as compared to the control (Dunnett's test, *p* < 0.002). Atrazine treatment showed similar effect to Roundup on the CP. We observed 41-68 % higher CP levels in *Daphnia* exposed to 0.1, 0.5, 1 and 5 μg/L of Atrazine as compared to the control group (Figure 2D(Fig. 2); Dunnett's test, *p* < 0.03). Probably, the treatment of *Daphnia* with these concentrations of herbicides promotes overproduction of free radicals resulting in oxidative damage of proteins.

**Figure 3:**
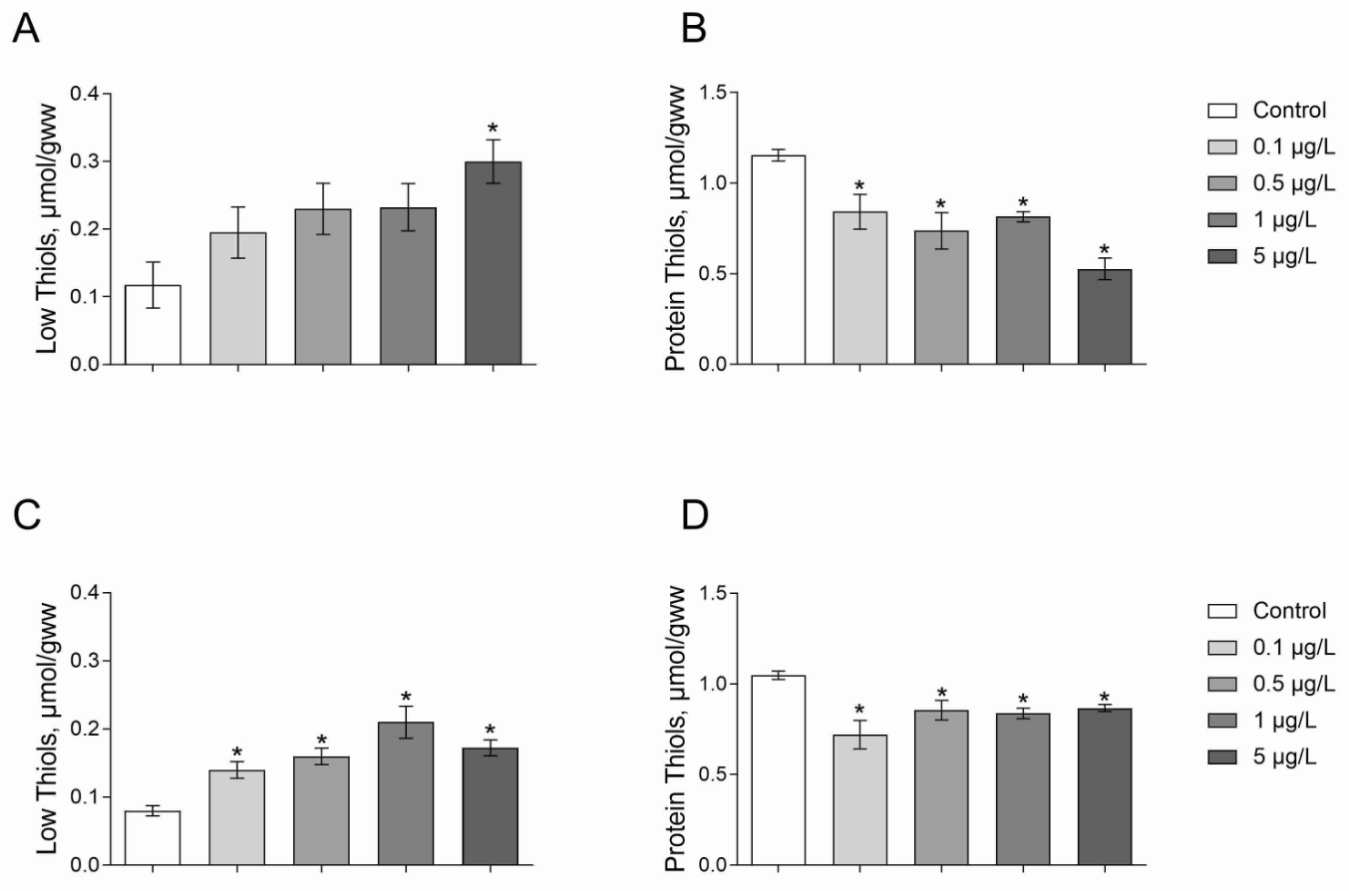
The levels of low-molecular-mass thiols (A, C) and protein thiol groups (B, D) in 7-8-day-old *D. magna* exposed to Roundup (A, B) or Atrazine (C, D) for 24 hours. Values are expressed as mean ± S.E.M. (n = 4-6). *Significantly different with *p* < 0.05 (ANOVA followed by Dunnett's multiple comparison tests)

The content of low-molecular-mass thiol-group containing compounds (L-SH) was 1.5-fold higher under the Roundup exposure at a concentration of 5 μg/L as compared to the control group (Figure 3a(Fig. 3); Dunnett's test, *p* = 0.013). Moreover, *Daphnia* exposed to Atrazine at all tested concentrations had significantly higher levels L-SH than those in control treatments (Figure 3C(Fig. 3); Dunnett's test, *p* < 0.04).

The study showed a reduced level of protein thiols (P-SH) under Roundup exposure. Indeed, we observed 28-57 % decreased content P-SH under Roundup treatment at the concentrations of 0.1-5 μg/L as compared to the control group of *Daphnia* (Figure 3B(Fig. 3); Dunnett's test, *p* < 0.03). The level of P-SH increased 17-31 % relative to control animals under exposure to Atrazine at a range of concentrations of 0.1-5 μg/L for 24 hours (Figure 3D(Fig. 3); Dunnett's test, *p* < 0.05).

### Activities of antioxidant enzymes

**Figure 4:**
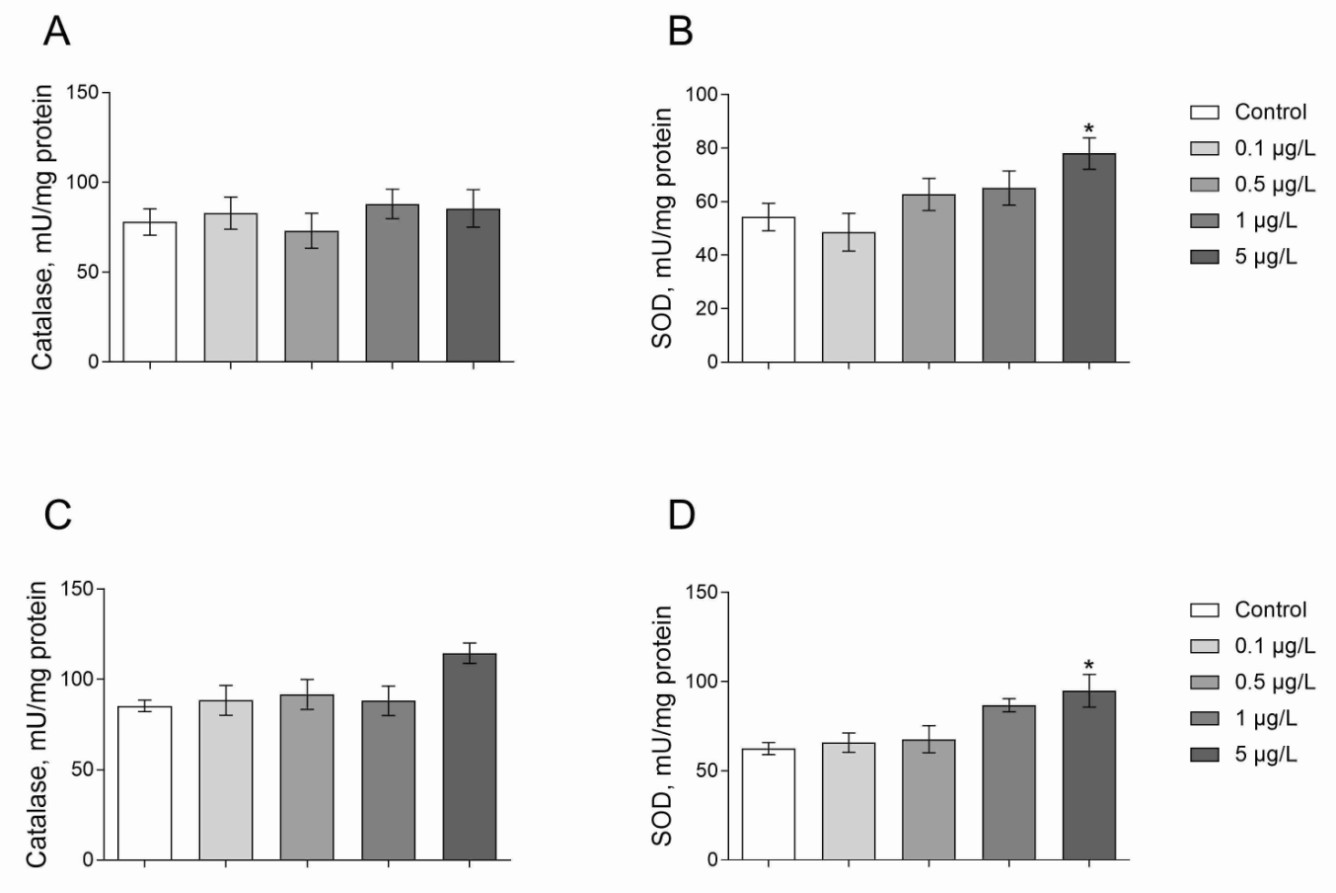
Catalase and SOD activities in *D. magna* exposed to Roundup (A, B) or Atrazine (C, D) for 24 hours. Values are expressed as mean ± S.E.M. (number of pools n = 3-6). *Significantly different with *p* < 0.05 (ANOVA followed by Dunnett's multiple comparison tests)

Superoxide dismutase (SOD) and catalase are considered as the most important antioxidant enzyme systems in invertebrate species. We observed 43 % higher SOD activity in Roundup exposed *Daphnia* at 5 μg/L for 24 h when compared with control group animals (Figure 4B(Fig. 4); Dunnett's test, *p* = 0.048). *Daphnia* exposed to Atrazine at concentrations of 1 and 5 μg/L had greater SOD activities by 41 and 49 % respectively as compared to control (Figure 4D(Fig. 4); Dunnett's test, *p* < 0.05).

The catalase activity in *Daphnia* of the control group was 78.0 ± 6.5 U/mg protein (A) and 85.2 ± 7.1 U/mg protein (B) (Figure 4(Fig. 4)). Catalase activity was not affected by both Roundup and Atrazine treatments for 24 hours at a range of concentrations of 0.1-5 μg/L (Figure 4(Fig. 4)).

## Discussion

The study was initiated to determine the acute toxicity of the formulated herbicides Roundup and Atrazine to the aquatic invertebrate *Daphnia magna*. Since many pesticides exert toxic effects related to oxidative stress, we also investigated biochemical responses of oxidative stress against Roundup or Atrazine and their toxicity consequences. In this study, *D. magna* showed LC_50_ under Roundup treatment for 24 hours at a concentration of 0.022 mg/L and 48 hours at a concentration of 0.0008 mg/L. Our results are in good agreement with the previous experimental data of Sarıgül and Bekcan (2009[44]) that showed that the LC_50_ for *D. magna* was 0.019 mg/L of Roundup (0.012 mg/L-0.024 mg/L 95 % confidence interval) for 24 hours, but the LC_50_ was 0.012 mg/L (0.001 mg/L-0.016 mg/L 95 % confidence interval) for 48 hours (Sarıgül and Bekcan, 2009[44]). We also found complete mortality at concentrations of 30 mg/L and 10 mg/L during 24 and 48 hours of Roundup exposure, respectively. A comparative study of the acute toxicity of six different glyphosate-based herbicides on *D. magna* found an LC_50_ value at the range of 4.2-117 mg/L (Melnichuk et al., 2007[32]). Various environmental tests of active glyphosate-based toxic substances significantly differ in LC_50_ value for *D. magna*. One study has reported LC_50_ values at 13-20 mg/L (FAO, 2001[11]), while others have reported LC_50_ at 234 mg/L (Le et al., 2010[22]), 780 mg/L (McAllister and Forbis, 1978[31]), 930 mg/L (Forbis and Boudreau, 1981[12]) or even more 200 mg/L (Pereira et al., 2009[41]).

We found that Atrazine is less toxic for *Daphnia* as compared to Roundup. The toxicity test showed LC_50_ at a concentration of 40 mg/L and 7 mg/L for 24 and 48 hours of Atrazine exposure respectively. Previously obtained LC_50_^48^ values for Atrazine were 24.6 mg/L (Phyu et al., 2004[42]), 26.9 mg/L and 36.5 mg/L for *D. carinata, D. magna* and *D. pulex*, respectively (Brooks, 1992[6]). We also found that exposure at the concentrations of 100 and 50 mg/L of this herbicide resulted in the total mortality of the *Daphnia* population within 24 hours and 48 hours, respectively.

As the consequence of the intensive use of Roundup, the glyphosate or the degrading product aminomethyl phosphonic acid (AMPA) are often detected in the aquatic ecosystems. The levels of surface water contamination by pesticides vary greatly in different countries depending on season (peak in spring/summer). Indeed, in the surface water of Netherlands the concentration of glyphosate is 0.5-1 μg/L and AMPA - 6 μg/L (IPCS, 1994[18]). Glyphosate concentration in the lakes and rivers is < 5153 μg/L (IPCS, 1994[18]), surface waters - 100-300 μg/L (Gottrup et al., 1976[15]) of Canada. In USA, the glyphosate concentration is 35-1237 μg/L and AMPA concentration is 2-35 μg/L (IPCS, 1994[18]). In Ukraine, there are no data on the content of glyphosate and AMPA in surface waters. The maximum permissible concentration of glyphosate in environmental waters is 0.02 mg/L. In many countries, after application in agricultural areas, Atrazine has been found in groundwater at levels of 0.01-6 µg/L. It has also been detected in drinking-water in several countries at levels of 0.01-5 µg/L (UNEP, 1998[49]).

The toxicity of pesticides could be also assessed using some biochemical parameters. Since pesticides could induce oxidative stress, we suggest measurements of oxidative damage levels for toxicity evaluation in *Daphnia*. Reactive oxygen species (ROS) damage almost all cellular macromolecules including proteins, lipids, nucleic acids. Cellular proteins are primary targets for ROS attack (Lushchak, 2011[27]). Various ROS and their derivatives cause specific types of damage to individual amino acids in the polypeptide chain via the formation of additional carbonyl groups. Hence, the determination of protein carbonyl content serves as a reliable indicator for the assessment of oxidative damage of proteins within the cells. In this study, it was found higher CP at all experimental concentrations of either Roundup or Atrazine. Hence, the treatment of *Daphnia* even at very low concentrations of these herbicides promotes ROS generation and the body's antioxidant system is not able to neutralize them, resulting in oxidative damage of proteins. The formation of protein carbonyl groups is a virtually irreversible process that is associated with a functional decline of the target proteins, which are thought to contribute to the aging process and age-related pathogenesis (Lushchak, 2011[27]).

Nowadays, a number of research have been devoted to studying the role of lipid peroxidation (LOOH) in the mechanisms of pathological conditions development within the body (Lushchak et al., 2004[26]). Lipid peroxidation is a process of free radical attack of lipids containing carbon-carbon double bond(s), especially polyunsaturated fatty acids. Accumulation of the LOOH products is associated with the complete destruction of unsaturated fatty acids and acetyl residues of phospholipids, disruption of the structure and function of proteins, the death of body cells. Lipid peroxidation is currently considered as one of the main causes of cell damage and death (Liu et al., 2013[24]; Pereira et al., 2009[41]). The results obtained for LOOH level indicated that Roundup and Atrazine exposure decreased the lipid peroxidation in *Daphnia*. However, previous studies have shown an increase in lipid peroxidation resulting from both pesticides treatment. Indeed, Blahová and colleagues (2013[4]) reported an increase in oxidized lipids in the kidneys of zebrafish (*Danio rerio*) exposed to Atrazine (30 and 90 μg/L) compared to controls (Blahová et al., 2013[4]). Independently, Chromcova and colleagues (2013[7]) and Nwani and colleagues (2010[34]) have also reported an increase in LOOH level due to the Atrazine exposure of various fish species. According to previous experimental data, Roundup exposure also led to an increase in the levels of reactive thiobarbituric acid (TBA-active products) in the silver carp tissues, which are thought to reflect the intensity of lipid peroxidation (Lushchak et al., 2009[25]). Lipid hydroperoxides are not only a marker for oxidative damage to lipids, they are also involved in the activation of antioxidant enzymes (Lushchak and Bagnyukova, 2006[29]; Valavanidis et al., 2006[50]). As a result, an enhanced antioxidant response can suppress the intensity of lipid peroxidation (Bagnyukova et al., 2007[1]).

Glutathione is involved in the detoxification of ROS through direct interaction with them or as a cofactor for antioxidant enzymes (glutathione peroxidase and glutathione-*S*-transferase) as well as systems for xenobiotics binding (Lushchak, 2012[28]). Oxidative stress, caused by xenobiotics, can deplete glutathione reserves and other low molecular weight thiol-containing compounds (Lushchak, 2011[27]). The level of thiol group-containing low molecular mass (L-SH) compounds was higher, but the level of protein thiols significantly decreased in Daphnia under 24-h Roundup and Atrazine exposure. Decreased levels of high molecular weight thiol groups of peptides and proteins may indicate irreversible destructive processes of these structures. Higher levels of thiol-containing compounds were previously found under a triazine pesticide exposure, namely simazine. It has been shown to increase glutathione levels in carp liver in a chronic experiment for 14 and 28 days. However, simazine exposure of fish for 60 days led to decreased glutathione content as compared to the control group of fish (Stara et al., 2018[45]). Oropesa and colleagues (2009[37]) observed an increased glutathione level in fish after acute exposure to simazine.

To minimize oxidative damage to cellular components, organisms have developed antioxidant defense systems. SOD is an enzyme of the first-line defense system against reactive oxygen species (ROS) which is responsible for the utilization of one of the most common free radicals - superoxide anion. Superoxide anion radical (O_2_˙ ¯) is perpetually generated in normal body metabolism, particularly through the mitochondrial energy production pathway. Therefore, superoxide dismutase activity is one of the markers of oxidative stress in the cell (Lushchak, 2011[27]). We found an increase in SOD activity under Roundup and Atrazine exposure for 24 hours in *Daphnia*. Higher SOD activity may indicate that the herbicide may cause superoxide anion overproduction, which in turn, serves as a signal to increase the activity of superoxide dismutase. Similar results were obtained in the study Jin and colleagues who reported an increase in SOD activity in the liver of zebrafish (*Danio rerio*) exposed to different concentrations of Atrazine for 14 days (Jin et al., 2010[20]). The activity of SOD was not affected in the brain, gills, liver and intestines of carp by Simazine exposure for 14, 28, or 60 days, however, SOD activity increased significantly in the muscles after 14 and 28 days of exposure to this herbicide (Stara et al., 2018[45]). However, Paulino and colleagues reported no changes in SOD activity in the gills in neotropic freshwater fish (*Prochilodus lineatus*) during acute exposure to different concentrations of atrazine (Paulino et al., 2012[39]). 

Catalase mitigates oxidative stress to a considerable extent by destroying cellular hydrogen peroxide to produce water and oxygen. Catalase is a constant component of cellular structures because it neutralizes hydrogen peroxide, which is a product of SOD. That is why catalase is considered as an additional oxidative stress marker (Bartosz, 2005[3]). However, we found that catalase activity was not affected by either Roundup or Atrazine exposure for 24 hours at 0.1-5 μg/L. Glusczak and co-authors (2007[13]) also did not show changes in catalase activity in the liver of *Rhamdia quelen* under Roundup exposure at concentrations of 0.2 and 0.4 mg/L for 96 hours. Catalase activity in the liver and kidneys of silver carp was higher under Roundup exposure for 96 hours as compared to the control and was not affected in the brain (Lushchak et al., 2009[25]). An increase in catalase activity was documented in the liver of *Prochilodus lineatus* which was exposed to 10 mg/L Roundup for 24 hours (Langiano and Martinez, 2008[21]). An increase in catalase activity due to the action of atrazine was observed in the other animal models. Indeed, Jin and colleagues (2010[20]) reported an increase in catalase activity in the liver of zebrafish after 14 days of exposure to Atrazine at a concentration of 1000 μg/L. Paulino and colleagues (2012[39]) reported no changes in catalase activity of gill in neotropic freshwater fish *Prochilodus lineatus* after acute exposure to different concentrations of Atrazine. However, triazine-induced inhibition of catalase activity was previously reported (Stara et al., 2018[45]). Blahova and colleagues (2013[4]) observed a significant decrease in catalase activity in all experimental groups of zebrafish exposed to Atrazine. Such differences in catalase activity indicated that it cannot be used as a reliable biomarker for Roundup toxicity. Although the activity of the catalase in *Daphnia* under Roundup treatment was not affected in current research, the hypothesis about potential Roundup involvement in ROS production, antioxidant defense responses and oxidative damage in aquatic invertebrates cannot be rejected.

Roundup concentrations that resulted in behavioral effects and induction of oxidative stress in the current study may be caused by the alteration of metabolic pathways via an effect on the gut microbiota in *Daphnia *(Suppa et al., 2020[48]). Roundup and its components appear to act through disruption of bioenergetic functions of mitochondria. Disruption of mitochondrial membrane potential is associated with high levels of reactive oxygen species and can be correlated with activation of caspases, which is harmful to a cell due to the high risk of apoptosis. Atrazine was shown to interfere *in vivo* life parameters by oxidative stress-induced retrogression and ecdysteroid biosynthetic pathway in *Tigriopus japonicas *(Yoon et al., 2019[52]). A significant increase in the intracellular ROS level was previously reported in *T. japonicus* in response to a high concentration (20 mg/L) of Atrazine (Yoon et al., 2019[52]). Many studies are indicating about atrazine induced oxidative stress in various aquatic animals (Falfushynska and Stolyar, 2009[10]; Jin, 2010[19]; Stara et al., 2012[46]). In this study, it has been found that the antioxidant enzyme SOD maintains a balance in cells of *Daphnia* and protects them from oxidative damage after exposure to Roundup and Atrazine at a range of concentrations 0.1-5 μg/L. Hence, some changes in antioxidant enzyme activities could occur as an adaptation mechanism to these stressful conditions. 

**Figure 5:**
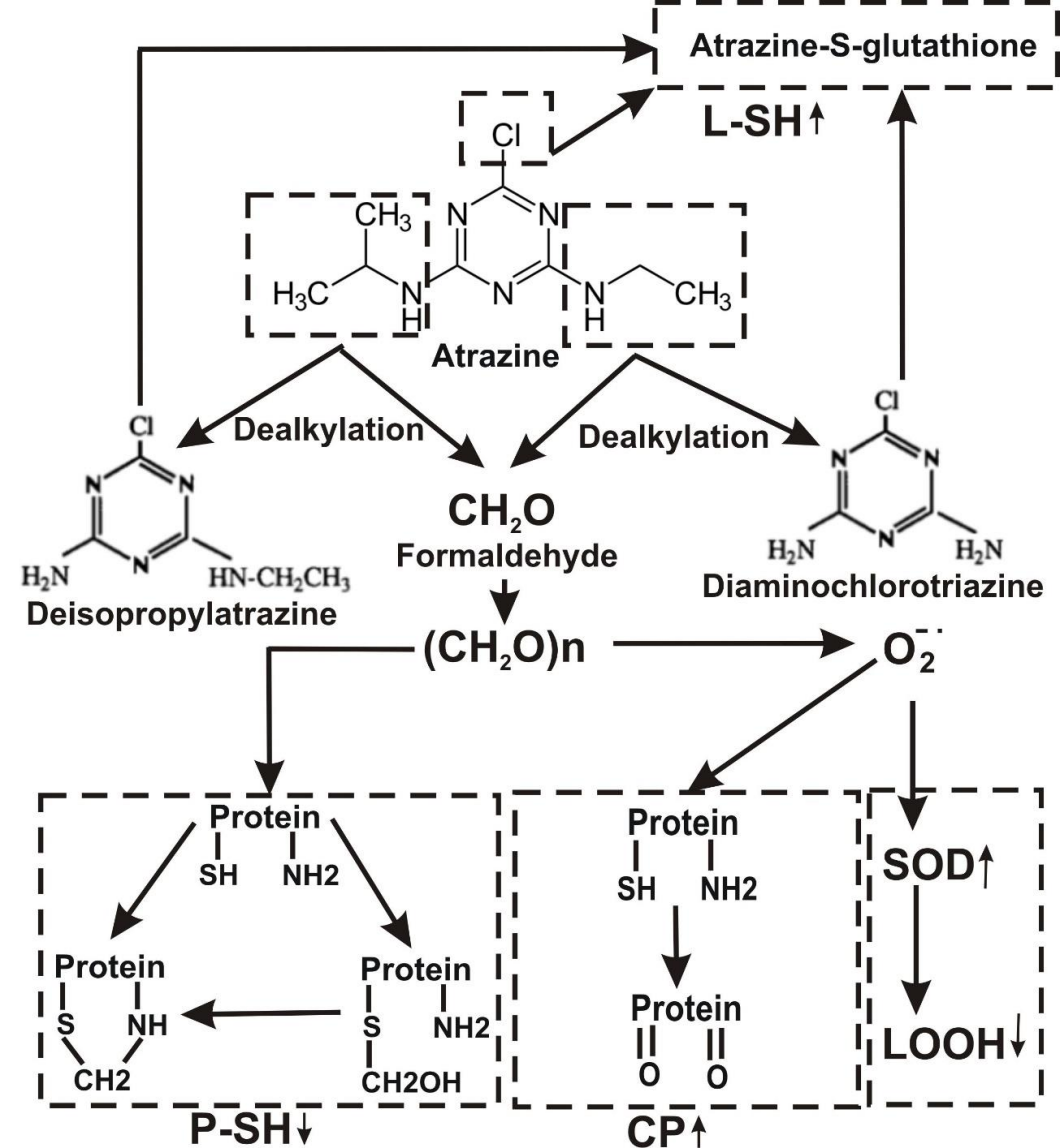
Hypothetical representation of mechanisms responsible for atrazine-induced stress in *Daphnia magna*. CP, carbonyl protein; L-SH, low molecular mass thiols; P-SH, high molecular mass thiols; LOOH, lipid peroxides; SOD, superoxide dismutase.

**Figure 6:**
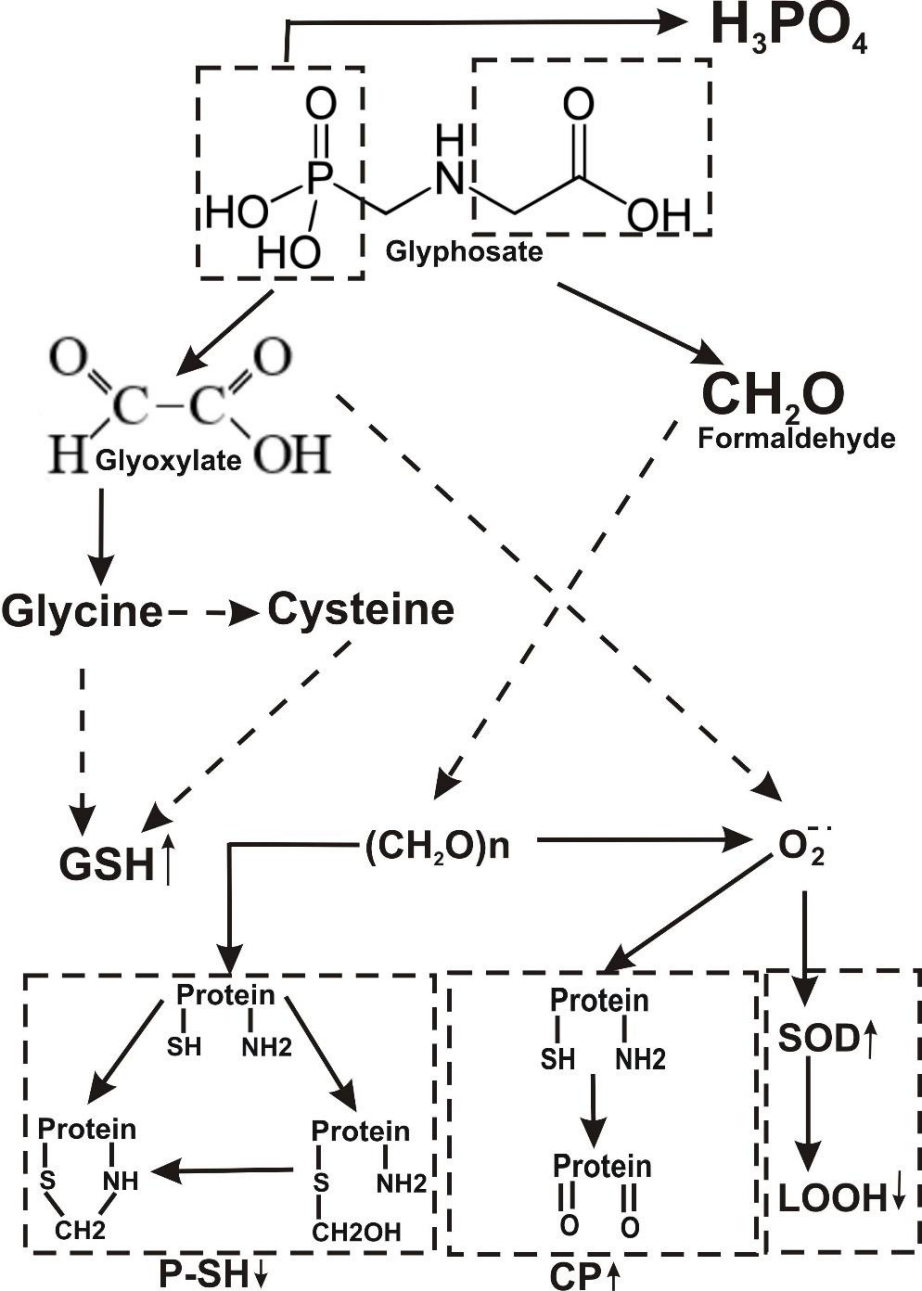
Hypothetical representation of mechanisms responsible for glyphosate-induced stress in *Daphnia magna*. CP, carbonyl protein; GSH, glutathione; P-SH, high molecular mass thiols; LOOH, lipid peroxides; SOD, superoxide dismutase.

The metabolism of a pesticide may play an important role in the determination of its toxicity. The major pathway of Atrazine decay involves alkyl-oxidation followed by dealkylation associated with formaldehyde formation as a by-product (Figure 5(Fig. 5)). The primary step involved in glyphosate degradation pathway is catalyzed by glyphosate oxidoreductase, which synthesizes the glyoxylate and AMPA. Degradation pathway of AMPA includes its breakdown to phosphonoformaldehyde via transaminase enzyme with subsequent transformation to formaldehyde (Figure 6(Fig. 6)). Meanwhile, the enhancement of the glyoxylate cycle contributed to the production of ROS.

## Conclusion

Overall, our results show that exposure to either Roundup or Atrazine causes embryonic developmental failure and causes contaminant-related induction of oxidative stress damage resulting from excessive ROS production in *Daphnia magna*. This is the first report that compares the effects of both herbicides using markers of oxidative stress and the activities of antioxidant enzymes. The common by-product of Atrazine and Roundup metabolism, formaldehyde, is considered to be contaminating chemicals that disturb free radical processes.

This study is important for understanding antioxidant response to the herbicides, the contribution of herbicides to *Daphnia *health, the risk assessment of pesticides to *Daphnia*, and reduction of pesticide threat to aquatic organisms. Since *Daphnia* is a key indicator of aquatic ecosystem health and serves as an important filter-feeder in aquatic ecosystems the glyphosate-based Roundup and triazine herbicide Atrazine can negatively influence water quality.

## Declaration

### Author contributions

Conceptualization, OL and HF; methodology, VH; validation, VH; formal analysis, VB; investigation, AP, VH; data curation, OL and VH; writing-original draft preparation, OS; writing-review and editing, OL and OS; supervision, NB and TS. All authors have read and agreed to the published version of the manuscript.

### Funding

This work was partially supported by the grant from the National Research Foundation of Ukraine #2020.02/0270.

### Institutional Review Board Statement

Not appicalble.

### Informed Consent Statement

Not applicable.

### Conflict of interest

The authors declare no conflict of interest.
